# The Determination of the Sexes

**Published:** 1881-03

**Authors:** 


### THE DETERMINATION OF THE SEXES.

Of the various theories that have been offered to explain the determination of sexes, the most plausible is that which assumes that the sex is determined by the relative maturity and vigor of the parent. When these qualities reside in the male, the offspring will probably be male, and *vice versa.* This view has some support from the fact that the proportion of birth (including still-birth) of males is to that of females as 143 to 100. Furthermore, the statistics of Hofacker and of Saddler corroborate the theory. According to the former, the proportion of male births to 100 females is as follows: father younger than mother, 90.6; father and mother of equal age, 90; father older by 1 to 6 years, 103.4; father older by 6 to 9 years, 124.7; father older by 9 to 18 years, 143.7; father older by 18 or more years, 200.

Saddler's statistics show very similar proportions. The actual proportion of living males to females at birth is, however, only about 105 to 100. The great losses of still-births, therefore, are among males, and are due chiefly to the larger size of the male child. The living male infant is about one-third of an inch longer and one-third of a pound heavier than the female. The average difference between the male and female fœtus is still greater. A large expenditure of valuable life, therefore, is produced by the pelvic strait being too small. Dr. Charles Roberts, who gives (*Lancet*) the above statistics, urges the necessity of a better physical education for girls, and intimates that the present practice of encouraging the intellectual development of women develops their crania at the expense of the pelvis, and leads to deplorable results. These results will be probably, in time, sterility, or, more probably, a lessened proportion of males. According to this philosophy, therefore, the enlargement of the pelvis should be one of the great goals of educational effort.

This is but a narrow view of the matter, however. There are many other factors in the question, and statistics so far show an excess of males in the most highly civilized races, as well as in those where woman is only a slave or a drudge.

